# Control of Nucleotide Metabolism Enables Mutant p53’s Oncogenic Gain-of-Function Activity

**DOI:** 10.3390/ijms18122759

**Published:** 2017-12-19

**Authors:** Valentina Schmidt, Rachana Nagar, Luis A. Martinez

**Affiliations:** Department of Pathology, Stony Brook University, Stony Brook, NY 11794, USA; valentina.schmidt@stonybrookmedicine.edu (V.S.); Rachana.Nagar@stonybrookmedicine.edu (R.N.)

**Keywords:** p53, nucleotide metabolism, GOFs, ETS2

## Abstract

Since its discovery as an oncoprotein in 1979, investigation into p53’s many identities has completed a full circle and today it is inarguably the most extensively studied tumor suppressor (wild-type p53 form or WTp53) and oncogene (mutant p53 form or mtp53) in cancer research. After the p53 protein was declared “Molecule of the Year” by *Science* in 1993, the p53 field exploded and a plethora of excellent reviews is now available on every aspect of p53 genetics and functional repertoire in a cell. Nevertheless, new functions of p53 continue to emerge. Here, we discuss a novel mechanism that contributes to mtp53’s Gain of Functions GOF (gain-of-function) activities and involves the upregulation of both nucleotide de novo synthesis and nucleoside salvage pathways.

## 1. Mutant p53’s Oncogenic Gain-of-Function

*TP53*, a gene which is located on chromosome 17p13.1 and encodes p53 protein, is more frequently mutated in various human tumors than any other gene in the genome [1]. A sequence-specific transcription factor, wild-type p53 (WTp53), has the distinction of being the first tumor suppressor protein identified [2]. It guards the organism by eliminating damaged cells mainly through the control of signaling pathways regulating DNA repair, cell-cycle progression, senescence, and apoptosis [3]. A role of WTp53 in control of cellular metabolism is also well-documented (reviewed in Reference [4]). However, mutations in *TP53* reverse the fate of p53 protein, granting it oncogenic powers, a phenomenon that is broadly referred to as “gain-of-function” or GOF [5]. The GOF of mutated p53 protein (mtp53) is manifested by its promotion of a multitude of cellular outcomes advantageous to a tumor cell, including cell growth and proliferation, invasion, metastasis, angiogenesis, genomic instability, somatic cell reprogramming, inflammation, and chemoresistance [6]. It is important to note that mtp53 also exerts its oncogenic properties through the inactivation of the tumor suppressive ability of WTp53, resulting in its loss-of-function (LOF). 

Since the 1989 discovery of a point mutation in the *TP53* gene [2], the intense debate continues on how p53 mutations can cause cancer beyond a mere inactivation of WTp53 function. Thousands of reports have established that mutations in the *TP53* gene are not restricted to colorectal cancer, where they were first identified, and now have been documented in >50% of all human tumors, although the p53 mutation spectrum and load vary between tumor types. Moreover, frequent tumor-associated p53 mutations are not restricted to humans, with mutations being identified in *TP53* orthologs of different organisms throughout the Animal Kingdom [3]. Unlike other tumor suppressor genes such as *APC* and *Rb1*, ~80% of *TP53* mutations represent missense mutations that result in amino acid substitutions in proteins. While these missense mutations have been detected in more than 250 codons of *TP53*, resulting in over 1800 different amino acid substitutions, they occur most frequently within the region encoding the DNA-binding domain of the p53 protein [7]. Comparative sequence analysis of p53 from different species pinpointed five highly conserved sequence blocks that match p53 mutation clusters found in human cancers. These blocks are referred to as “hotspot” mutations and most commonly affect R175, G245, R248, R249, and R273 so-called “hotspot” mutations in human tumors. Hotspot p53 mutations are prevalent in both sporadic tumors and familial Li-Fraumeni syndrome, a rare disorder characterized by predisposition to breast cancer, sarcomas, and other tumors [8,9,10]. A comprehensive review of *TP53* mutations can be found elsewhere [11,12,13,14]. 

Consequences of missense mutations include p53 protein conformational changes, increased protein stability, defective DNA binding, and transcriptional regulation. Mtp53 proteins are generally categorized as DNA contact (or class I) vs. conformational (or class II) mutants, depending on whether the mutation occurs in residues that make contact with DNA or disrupt the protein’s tertiary structure, respectively. Similar to the establishment of the tumor suppressive function of WTp53 earlier, various engineered knockout (KO) and knock-in (KI) mouse models recently were instrumental, in addition to in vitro tools, in defining GOF activities of various mtp53s [15,16,17]. Mouse models provide an independent proof of the mtp53 GOF concept, since mice harboring p53 mutants develop tumors distinct from those found in models lacking WTp53 (p53-null). The confirmed mechanisms of mtp53 GOF include the inhibition of p63- and p73-mediated transcription of WTp53 target genes through a physical interaction with p63 and p73 [18,19]; activation of oncogenic target genes through a direct interaction with their transcription factors; regulation of specific gene promoter transcription activities through a structure-dependent DNA binding; and a physical interaction with cytoplasmic proteins other than transcription factors [6,12]. Noteworthy is that, for a specific mtp53, these mechanisms are not necessarily mutually exclusive. There is also emerging evidence that the GOF activities of mtp53 are also context-dependent, with stromal alterations having a direct effect on tumor development [20]. 

In contrast to WTp53, mtp53-response DNA elements have not been identified to date and the different promoters activated by various p53 mutants share no sequence homology. However, both WTp53 and mtp53 have been reported to recognize DNA structural motifs in promoters including G-rich regulatory regions, supercoiled DNA, quadruplexes, and cruciforms [21,22,23,24,25]. There is also a possibility of mtp53 regulating gene expression via nuclear matrix attachment regions (MARs), since several tumor-derived mtp53s exhibit a high affinity for MARs [12]. Further discussion of the recognition of DNA structures by p53 can be found in the accompanying review by Brazda and Coufal [26]. Mtp53 has been shown to be active in both cytosol and the nucleus. Mtp53 nuclear activities are mostly attributed to transcriptional regulation (such as the transcriptional activation of PDGFRβ signaling in pancreatic cancer [27] or Pla2g16 phospholipase in osteosarcoma and mammary tumors [28]), as well as the regulation of the chromatin function [29]. 

Currently, the best experimentally supported model for the mechanism of the transcriptional GOF activity of mtp53 consists of mtp53 recruitment to target gene promoters via interaction with other proteins (transcription factors), which, in turn, tether mtp53 to promoters. NF-Y complex [30], SP1 [31], SREBP [32,33], VDR [34], ETS1 [35,36], and, most recently, ETS2 are the examples of such “escort” transcription factors. In the case of ETS2, such “oncogenic cooperation” [37] is mutually beneficial, since the binding to mtp53 stabilizes ETS2 and boosts its activity, subsequently triggering their downstream signaling programs. We recently reported that ETS2, a member of the evolutionally conserved erythroblastosis E26 transformation-specific (ETS) family of transcription factors and a downstream effector of the Ras/Raf/MAPK pathway, directly interacts with mtp53 [38]. We also showed that mtp53/ETS2 complex upregulates the expression of 5′-tyrosyl DNA phosphodiesterase TDP2, which is involved in the repair of DNA damage and promotes resistance to a chemotherapeutic agent etoposide [38]. Similarly, ETS2 appears to mediate mtp53 binding to the Pla2g16 promoter to induce free fatty acid synthesis and metastasis [28]. On a broader scale, by using both ChIP-on-chip and ChIP-seq analysis, this study identified the presence of the ETS site (GGAAR) within the predicted mtp53 binding motif in promoters of ~50% of various mtp53 target genes [38]. Notably, all conformational and DNA contact p53 mutants tested have been shown to interact with ETS2, albeit with different affinity [29,38]. Our most recent study demonstrated that mtp53 also protects ETS2 from ubiquitin-dependent degradation by blocking the ubiquitin ligase components Det1 and COP1 from binding to ETS2 [39]. Collectively, this emerging evidence suggests a novel ETS2-dependent mechanism for diverse mtp53’s GOF activities and warrants further investigation [40]. 

## 2. Nucleotide Metabolism and Its Regulation

Mtp53 heavily relies on the reprogramming of various aspects of cellular metabolism (reviewed in Reference [41]) for its GOF activities. These include the induction of the Warburg effect by an increase in glucose uptake, realized through the activation of RhoA and promotion of GLUT1 translocation to the plasma membrane [42], control of phospholipid and fatty acid metabolism [28], alteration of energy metabolism via the inhibition of AMPK [43], and steroid synthesis through the upregulation of the mevalonate pathway [32], among others. A tight control of various cellular and mitochondrial metabolic pathways is also an important component of the tumor suppressive function of WTp53 [44].

Rapidly proliferating cancer cells require a high turnover of RNA and therefore greatly depend on efficient nucleotide biosynthesis, which they often reprogram. In addition to being subunits for the nucleic acid synthesis, nucleotides also serve as coenzymes in a variety of metabolic reactions, and therefore the control of nucleotide levels is fundamental to proper cellular function. Nucleotide biosynthesis is an energy-intensive process utilizing multiple metabolic pathways, different cell compartments, and several sources of carbon and nitrogen. An array of multifunctional enzymes is involved in this complex process. Various enzyme activities are tightly regulated at different stages of the cell cycle at two levels: at the gene transcription level by a growing set of master transcription regulators and at the enzyme level by allosteric regulation and feedback inhibition. Yet, despite its functional importance, nucleotide metabolism is often overlooked by mtp53 investigators in favor of functional genomics. The significance of nucleotide metabolism for therapy is underscored by a number of drugs developed over the years for various maladies, such as nucleoside analog gemcitabine [45], purine analogs [46], pyrimidine analog fluorouracil (5-FU) [47], and antiviral nucleoside and nucleotide analogs [48]. 

**Figure 1:**
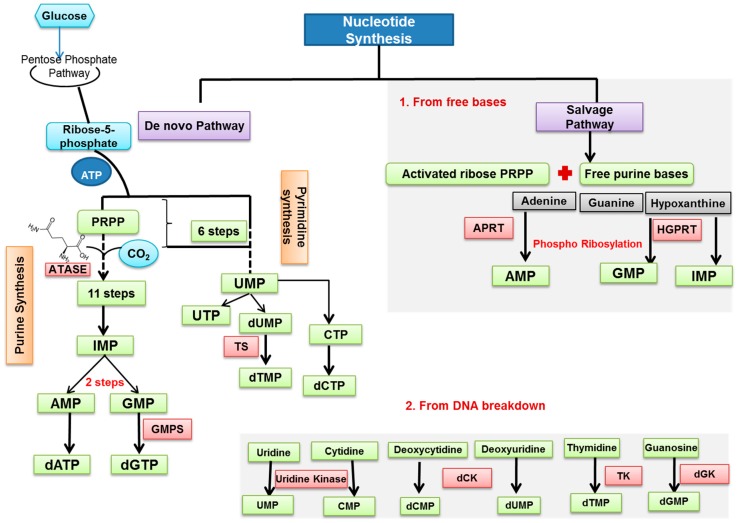
An overview of nucleotide synthesis. PRPP = 5-phospho ribosyl pyro phosphate; ATASE = amidophosphoribosyltransferase; IMP = inosine mono phosphate; AMP = adenosine mono phosphate; GMP = guanosine mono phosphate; CMP = cytidine mono phosphate; GMPS = guanosine mono phosphate synthetase; UMP = uridine mono phosphate; TS = thymidine synthetase; dATP = deoxyadenosine tri phosphate; dGTP = deoxyguanosine tri phosphate; dCTP = deoxycytosine tri phosphate; dTMP = deoxythymidine mono phosphate; APRT = adenine phosphoribosyl transferases; HGPRT = hypoxanthine-guanine phosphoribosyl transferases; dCK = deoxycytidine kinase; dGK = deoxyguanosine kinase. The dotted line represents the multiple steps.

In the cell, nucleotides can be synthesized either through the de novo or the salvage pathways [49] (Figure 1). The metabolic demands of nucleic acid synthesis have been reviewed in Reference [50]. The de novo pathway relies on glucose, glutamine, and CO_2_ for building materials: glucose is converted to ribose-5-phosphate (ribose-5-P) and glutamine serves as a source of nitrogen. Ribose-5-P, a product of the pentose phosphate pathway (PPP), is used for both purine and pyrimidine syntheses. Glucose-6-phosphate dehydrogenase (G6PD) is the rate-limiting enzyme of the PPP pathway. Aside from the utilization of ribose-5-phosphate, purine and pyrimidine synthesis follow divergent pathways. Purines take 11 steps to build on the ribose-5-P backbone and produce inosine monophosphate (IMP), which, in turn, is modified to finally produce adenosine monophosphate (AMP) and guanosine monophosphate (GMP). GMP synthetase (GMPS) is one of three glutamine amidotransferases involved in de novo purine biosynthesis and is responsible for the last step in the synthesis of the guanine nucleotide. In contrast to purine synthesis, it takes the cell only six steps to synthesize the pyrimidine ring, with uridine monophosphate (UMP) as a final product. UMP then can be converted into cytidine triphosphate (CTP). Thymine nucleotides are synthesized via the reduction of uridine diphosphate (UDP) and cytidine diphosphate (CDP), and thymidylate synthase (TS) is responsible for dTTP synthesis [49].

The salvage pathway, which works in cytosol and mitochondria, is an alternative to the de novo pathway for the synthesis of both purines and pyrimidines, which recycles purine and pyrimidine rings available in the cell upon the turnover and degradation of cellular material (Figure 1). While de novo synthesis is evolutionally conserved, salvage pathways show significant diversity. Free purine bases can be converted to corresponding nucleotides through phosphoribosylation utilizing 5-phosphoribosyl-1pyrophosphate (PRPP). The two key transferases responsible for this reaction in mammals are adenine phosphoribosyltransferase (APRT, mediates the transfer of PRPP to adenine to form AMP) and hypoxanthine-guanine phosphribosyltransferase (HGPRT, mediates the formation of IMP and GMP). Interestingly, pyrimidine salvage is more efficient than purine salvage [49]. Nucleosides are also anabolized by kinases. Deoxyribonucleoside kinases found in humans include deoxycytidine kinase (dCK), which is the principal enzyme for the utilization of purine deoxyribonucleosides, two thymidine kinases (TK1 and TK2), and deoxyguanosine kinase (dGK) [51]. These kinases display distinct intracellular distribution, with dCK and TK1 being restricted to cytosol, and TK2 and dGK to mitochondria. While most somatic cells possess de novo nucleotide synthetic capabilities, the salvage enzymes are usually not required for cell viability. Exceptions include T and B lymphocytes. dCK−/− mice displayed a 90-fold decrease in thymic cellularity and a 5- to 13-fold decrease in lymphocyte numbers compared to wild-type littermate controls due to replication stress (RS) and DNA damage, indicating that the deoxyribonucleoside salvage pathway is required for normal lymphocyte development [52]. Also, while it is generally accepted that proliferating cells favor the de novo nucleotide biosynthesis pathway, it has been discovered recently using a computational systems approach and metabolomics data from a cohort of colorectal carcinoma (CRC) patient tumors that CRC cells utilize the salvage pathway in parallel with the de novo pathway for purine synthesis [53]. In particular, the expression of three enzymes involved in nucleotide biosynthesis has been shown to be altered in CRC to accommodate its demand for increased nucleotide levels. The activity of amidophosphoribosyltransferase (ATASE), a rate-limiting enzyme of the first step of the de novo pathway that catalyzes the PRPP to IMP conversion, was elevated 2-fold in CRC tumors compared to normal tissue controls. On the other hand, significant downregulation in CRC tumors of 5′-nucleotidase (5NUC), a plasma membrane enzyme that converts extracellular nucleotides to the corresponding nucleosides to aid their entry into the cell, and xanthine oxidase/dehydrogenase (XD), which competes for substrate with HGPRT, is consistent with elevated activity of the salvage pathway [53]. Another emerging aspect of the salvage pathway is that salvaged nucleosides often contain epigenetic modifications and can be damaging to the cell if incorporated into DNA. It has been recently shown that enzymes of the nucleotide salvage pathway display remarkable substrate selectivity, effectively protecting newly synthesized DNA from the incorporation of modified forms of cytosine, such as 5-methyl-2′-deoxycytidine (5mdC) [54].

The cell requires a balanced supply of a particular type of nucleotide, 2′-deoxyribonucleoside 5′-triphosphate (dNTP), to replicate and repair its DNA properly. Ribonucleotide reductase (RNR) catalyzes the reduction of all four NDPs or NTPs at the 2′ position of ribose sugar to dNTP or dNTP, a rate-limiting step in dNTP synthesis [55]. Nicotinamide adenine dinucleotide phosphate (NADPH) provides electrons. RNR is a tightly regulated tetrameric enzyme consisting of two catalytic subunits (RRM1) and two regulatory subunits (RRM2 and p53R2/RRM2B) [49,55]. The RRM2 subunit controls the reduction during the S phase of the cell cycle and supplies dNTPs for DNA replication [56], while p53R2 is involved in supplying dNTPs for DNA repair and mitochondrial DNA synthesis in the G0/G1 phase of the cell cycle [57]. Recent evidence shows that RRM2 plays a key role in the oncogene-induced senescence (OIS) mechanism and introduces nucleotide metabolism as an important new pathway regulating OIS [58]. If RRM2 is not downregulated, cells with activated oncogenes can escape senescence and undergo oncogenic transformation [58]. Under normal growth conditions, RRM2 expression is controlled by E2F transcription factor 1 (E2F1) [59]. When E2F transcription factor 7 (E2F7), a transcriptional repressor, replaces E2F1 at the *RRM2* gene promoter region, the downregulation of RRM2 expression occurs [60]. In turn, the downregulation of RRM2 activates WTp53 [60] and, consequently, E2F7 [61], indicating the existence of a negative feedback loop where a decrease in RRM2 leads to its further reduction via the activation of the p53/E2F7 and Rb pathways. Remarkably, senescence caused by RRM2 inhibition in melanoma and ovarian cancer cells is independent of both p53 and Rb [60,62]. While OIS is a cellular tumor suppressor mechanism aimed to permanently halt cell cycle progression, a decreased dNTP pool caused by oncogene-induced downregulation of RRM2 triggers replication stress and accumulation of DNA damage prior to the onset of senescence [60,63,64]. This is in agreement with an earlier study by Bester et al. [65] demonstrating that insufficient nucleotide synthesis is responsible for DNA damage and genome instability caused by the activation of the Rb-E2F oncogenic pathway. Importantly, the deprivation of the micronutrient folate, which is required for multiple nucleotide biosynthesis steps, enhanced oncogene-induced replication stress, DNA damage, and oncogenic transformation [66]. Thus, the oncogene-dependent promotion of cell growth in the context of an inadequate amount of nucleotide synthesis to support high-fidelity DNA replication may contribute to the acquisition of mutations that lead to the bypass of cellular senescence and progression towards oncogenic transformation. 

## 3. Control of Nucleotide Metabolism by Mtp53

Both tumor suppressors (e.g., *WTp53*, *Rb*) and oncogenes (e.g., *E2Fs* and *myc*) have been identified as transcriptional regulators of nucleotide metabolism [63,67,68,69]. Genes encoding nucleotide biosynthesis enzymes span nine different chromosomes in humans, and the expression of these genes is controlled by master transcription factors [70]. WTp53 has been implicated in the transcriptional regulation of several enzymes from the purine de novo synthesis pathway (phosphoribosyl pyrophosphate synthetase or PRPS, phosphoribosyl aminoimidazole carboxylase or PAICS, GMP synthetase or GMPS, and adenylate kinase or AK1), along with enzymes involved in ribose synthesis (glucose-6-phosphate dehydrogenase or G6PD, transketolase or TKT) and various feeder pathways (phosphoserine phosphatase or PSPH, thymidylate synthetase or TYMS, dihydrofolate reductase or DHFR, and ribonucleotide reductase subunit 1 or RRM1) [70]. Wtp53 can also temporary activate ribonucleotide reductase subunit 2 (RRM2) in response to DNA damage [71,72] as a part of the feedback loop described earlier. These regulatory mechanisms change when p53 acquires mutations and becomes a constitutive activator of RRM2.

Our recent work using ChIP analysis on MDAMB231 and MiaPaCa-2 cells revealed that mtp53 associates with promoters of multiple nucleotide metabolism genes (NMG) [73]. Among them was the gene encoding RRM2 subunit (*RRM2b*). We have demonstrated that mtp53 binds the *RRM2b* gene promoter sequence [73], while, in contrast, WTp53 is known to regulate *RRM2b* through interaction with its first intronic region ~1.5 kb downstream from the promoter [71]. These data agree with our previous observation that mtp53- and WTp53-regulatory binding sites do not overlap [38]. Also, our findings are in agreement with the report by Nakano et al. [72], in which they showed that both WTp53 and mtp53, namely R175P and V143A (but not R175H), were able to induce expression of the ribonucleotide reductase subunit p53R2. The p53 mutants found to activate RRM2 in our study included R249S, R273L, R273H, R280K, and R248W [73]. Yet, Tanaka et al. [71] reported that mtp53 was unable to activate p53R2 expression, although it is unclear which specific point mutation they studied. This points out a possibility that various p53 mutants may have highly specialized functions in respect to the NMG regulation. 

Furthermore, our studies beyond RRM2 demonstrated that mtp53 transcriptionally activates the expression of many other NMG (such as *dCK*, *TK1*, *GMPS*, *IMPDH1*, *PAICS*) involved in both the de novo nucleotide synthesis and salvage pathways and hence controls the nucleotide pools in the cell [73]. RNAi-based experiments confirmed that NMG expression levels were directly linked to the levels of mtp53, while the depletion of serum (i.e., removal of mitogens) in culture media proved that the observed decrease in NMG expression upon mtp53 knockdown is a direct consequence of loss of mtp53 GOF and not just a result of reduced cell proliferation. 

This study also discovered a new mtp53 GOF—creating a dependence on dCK in mtp53-harbouring cells [73]. *dCK* was found among NMG upregulated by mtp53. We speculated that the upregulation of multiple NMG by mtp53 caused elevated levels of dTTP, which in turn allosterically inhibited the production of dCDP, thus making the cells dependent on the production of dCTP through the nucleoside salvage pathway. A long-term (>1 week) propagation of breast carcinoma cells lines with dCK knockdown revealed a significant reduction in their proliferation rates; however, the same dCK knockdown in normal human mammary epithelial cells (HMEC) or lung fibroblasts did not affect cell proliferation [73]. This implies that mtp53-harboring cells acquire a synthetic sick or synthetic lethal phenotype and become dCK-dependent. 

Next, since the previous study by our group identified the ETS binding site in promoters of various mtp53 target genes [38] and the majority of NMG display ETS-binding motifs in their promotors, an effect of both ETS1 and ETS2 on mtp53-mediated NMG expression was tested. It was determined that ETS2, and not ETS1, regulated NMG expression in concert with mtp53 [73]. Importantly, the transcriptional control of the NMG exerted by mtp53 in the breast cancer cell lines studied was shown to be sufficient to impact the rNTP and dNTP pools, and, consequently, the activity of GTP-binding GTPases (such as Ras, Rac1, and Cdc42) and cell invasiveness in several breast and pancreatic cancer cell lines [73]. GMPS, which catalyzes the final steps of the de novo nucleotide synthesis pathway, resulting in GMP production, was shown to be required to sustain an invasive phenotype of breast carcinoma in our in vitro and mouse xenograft experiments. Moreover, the significance of mtp53 control of the salvage nucleotide synthesis pathway was underscored by the discovery that the induction of dCK kinase by mtp53 and ETS2 sensitized cells to nucleoside analogs commonly used as chemotherapy agents [73]. In contrast, it was reported previously by the Chumakov group that mtp53-mediated induction of UTPase, an enzyme involved in thymine de novo synthesis, conveys 5-FU resistance [74]. Finally, data mining of 537 breast cancer patient transcriptomes revealed that 11 out of 16 NMG analyzed were upregulated in tumors containing mtp53. The elevated expression of these NMGs correlated with poorer prognosis for relapse-free survival (RFS) and distant metastasis-free survival (DMFS) [73]. 

In addition to being important for proliferation, nucleotides and nucleosides can mediate intra and inter-cellular signaling [75,76]. In the tumor microenvironment, nucleotides and nucleosides can mediate the crosstalk between tumor cells, immune cells, and stroma via the activation of purinergic receptors. This is highly significant since activation of the purinergic receptors has been shown to promote the epithelial to mesenchymal transition and to promote invasion [75,77,78]. Furthermore, extracellular nucleotides and nucleosides can be immunostimulatory or immunosuppressive depending on the microenvironment, and the activation of purinergic receptors has been shown to protect cancer cells from antitumor T cells [76,79,80]. Importantly, extracellular concentrations of nucleotide and nucleosides can be controlled by a family of cell-surface associated enzymes called ectonucleotidases [81]. A recent study by the Stiewe group identified the ectonucleotidase ENTPD5 as a transcriptional target of mtp53 [82]. Although ENTPD5 has been shown to be secreted from cells, it is also found in the endoplasmic reticulum [83,84]. ENTPD5 was found to mediate the pro-invasive activities and lung metastasis driven by mtp53 via its role in promoting *N*-glycoprotein folding and maturation [82]. To what extent the extracellular ectonucleotidase activity of ENTPD5 contributes to mtp53’s GOF remains to be seen. Apart from the Chumakov group’s report on the ability of mtp53 R273H to regulate the key enzymes in the nucleotide biosynthesis salvage pathway [85], the study from our group [73] is the first comprehensive work implicating mtp53 in the control of NMG, and hence adds another contribution to the already sprawling mtp53 GOF repertoire. Significantly, the expression of oncogenic myc, a known regulator of NMG expression [63] and a target of mtp53 transcriptional activity [86], was not affected by mtp53 knockdown [73], eliminating the possibility that mtp53 indirectly induces NMG expression by upregulating myc. We demonstrated that several distinct p53 mutants were capable of regulating NMG expression, emphasizing that this new GOF is a common mtp53 feature. However, previous in vivo studies have shown that the degree of GOF induced by different mtp53 often varies [17], and more studies are needed to dissect the individual mtp53 contribution to NMG regulation. 

As described above, we recently showed that ETS2 tethers mtp53 to the promoters of its numerous targets and therefore assists mtp53 in the activation of genes. Knockdown of either ETS2 or mtp53 reduced the expression of NMG [73], which suggests that the mtp53/ETS2 complex is needed for the optimal control of NMG expression. The more intricate aspects of mtp53/ETS2 interaction and its consequences for mtp53 GOF are reviewed in Reference [40]. The Lozano group previously showed that the mtp53/ETS2 complex induced the migration and invasiveness of osteosarcoma cells by the upregulation of phospholipase Pla2g16 expression [28]. In agreement, we found that transient knockdown of mtp53 was sufficient to reduce cell proliferation and invasion, in addition to NMG expression [73]. It is well documented in vitro and in vivo that perturbations in nucleotide metabolism not only impact proliferation but also invasion and metastasis [58]. Thus, decreased expression of guanosine monophosphate reductase (GMPR) increases guanosine triphosphate (GTP) levels, which drives melanoma invasion [87]. This is due to the fact that GTP is a crucial component for the activation of GTPases, master signal transducers. Active (GTP-bound) forms of Ras and Rho GTPases drive cell proliferation, migration, and oncogenic transformation [88]. Noteworthy, in addition to the control of nucleotide levels, mtp53 may also be able to exert its GOF and mediate GTPases activity through their corresponding GTPase-activating proteins [89]. Our study also revealed that GMPS knockdown reduced GTP levels and blocked the development of breast cancer brain metastatic lesions in vivo [73], which is in line with Reference [87]. Chen et al. [90] reported that in order to metastasize to the brain, breast and lung cancer cells establish carcinoma-astrocyte gap junctions and use the connexin 43 (Cx43) channels and PCDH7 neuronal receptors to transfer the second messenger cGAMP to astrocytes to create a favorable environment (i.e., a niche) for carcinoma cells to grow by activating their STAT1 and NF-κB survival signals [90]. When we knocked down GMPS, an enzyme upstream from cGAMP in the salvage nucleotide synthesis pathway, in MDA231-BrM cells, the cells with depleted GMPS were unable to form brain metastasis in vivo [73]. We speculate that in our system, the inactivation of GMPS potentially prevented the generation of cGAMP and this, in turn, resulted in the absence of a “carcinoma-friendly” environment in brain astrocytes and therefore protection from metastatic lesions. 

Interestingly, the upregulation of purine synthesis has been implicated in the growth, self-renewal, and in vivo tumor formation of glioma brain tumor initiating cells [91]. In this study, MYC was implicated in the transcriptional induction of these purine synthetic enzymes [91]. Importantly, shRNA knockdown of the purine synthesis enzymes PRPS1 (phosphoribosyl pyrophosphate synthetase 1), ADSL (adenylosuccinate synthase), or GMPS strongly suppressed the development of glioblastoma tumors in immunocompromised mice [91]. As this study investigated the development of glioblastoma, the in vivo tumorigenesis assay involved the intracranial engraftment of cancer cells. It is worth noting that the metastatic process requires the invasion of adjacent tissues, entrance and transit through the circulatory system, and extravasation and colonization of the metastatic niche. Thus, in our study we cannot rule out the possibility that GMPS is required for multiple steps in the metastatic process that are required for eventual colonization [73]. However, in this glioblastoma study, the observation that GMPS knockdown prevents tumor growth supports the notion that GMPS may contribute to the establishment of the metastatic niche via crosstalk with neighboring cells. 

**Figure 2:**
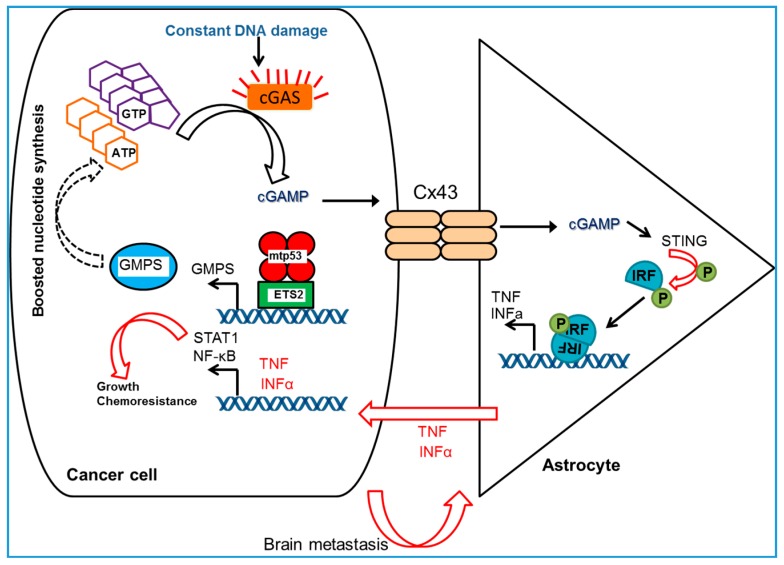
Schematic showing the mechanisms of cancer cells metastasis in brain. Adapted and modified from Chen et al. [90].

Based on the collective evidence presented above, we propose the following model for the mechanism of the mtp53-mediated regulation of metastatic invasion into the brain via control of the salvage nucleotide synthesis pathway (Figure 2). In a cancer cell, nuclear mtp53 tetramers bind ETS2 and, with its assistance, recognize and bind the promoter regions of numerous NMG, GMPS being one among them. Mtp53 then induces the transcription of GMPS, which results in the generation of an abundant supply of cGAMP messenger. cGAMP is transmitted into the neighboring astrocyte through gap junction Cx43 channels and triggers the activation of STING [90,92], an innate immune response pathway, as well as the production of inflammatory cytokines, such as INF-α and TNF. The cytokines activate STAT and NF-κB survival pathways back in the cancer cell, stimulating its growth and providing chemoresistance [90]. Given that many chemotherapeutic agents do not effectively cross the blood-brain barrier, the promotion of metastasis to the brain by mutant p53 may provide an indirect mode of protecting cancer cells from chemotherapy. Thus, by upregulating nucleotide synthesis, mtp53 may increase both cell growth and crosstalk between cancer cells and the neighboring cells that form the metastatic niche. 

## 4. Conclusions

Emerging evidence presented in this review identifies the control of nucleotide pools as an important component of mtp53 GOF activities, which validates further exploration. Multiple new mtp53 targets have been identified among NMG from both the nucleotide de novo synthesis and salvage pathways, and a proposed mechanism of the mtp53 control of brain metastasis through its NMG target GMPS is especially intriguing. This emphasizes that the individual tumor status of p53 may aid in the selection of chemotherapeutic agents used as first-line treatment. Targeted allele-specific silencing of mtp53 using RNAi technology has been shown to eliminate mutant-specific GOFs while sparing endogenous WTp53 activity in vitro and in vivo, thus representing a promising approach for cancer therapy [93,94], although delivery remains a major challenge for clinical applicability. Recently, pharmacological approaches have been used to destabilize the mtp53 protein and these approaches hold promise for directly targeting this oncogene [95,96]. In addition, an apparent addiction of mtp53-harboring cells to the nucleotide salvage pathway enzyme dCK may offer additional therapeutic opportunity since normal cells do not appear to be dependent on this enzyme. 

## Abbreviations

APC	APC, WNT signaling pathway regulator
RB1	RB transcriptional corepressor 1
PDGFRβ	Platelet-derived growth factor receptor β
NF-Y	Trimeric complex composed of NFYA, NFYB, NFYC
SP1	SP1 transcription factor
SREBP	Sterol regulatory element binding protein
VDR	Vitamin D receptor
ETS1	ETS proto-oncogene 1, transcription factor
ETS2	ETS proto-oncogene 2, transcription factor
GLUT1	SLC2A1, solute carrier 2, member 1
AMPK	PRKAA1, protein kinase AMP-activated catalytic subunit alpha 1
cGAMP	cyclic GMP-AMP
ENTPD5	Ectonucleoside triphosphate diphosphohydrolase 5
